# Periviability Decision-Making: Assessing Provider Characteristics and Cognitive Traits

**DOI:** 10.1089/whr.2021.0014

**Published:** 2021-06-29

**Authors:** Ariel Sklar, Amy Yang, Noelle G. Martinez, Lynn M. Yee

**Affiliations:** ^1^ Division of Maternal-Fetal Medicine, Department of Obstetrics and Gynecology, Northwestern University Feinberg School of Medicine, Chicago, Illinois, USA.; ^2^ Department of Obstetrics and Gynecology, Kaiser Permanente, San Leandro, California, USA.; ^3^ Biostatistics Collaboration Center, Northwestern University Clinical and Translational Sciences Institute, Northwestern University Feinberg School of Medicine, Chicago, Illinois, USA.

**Keywords:** decision-making, periviability, physician cognitive skills, physician coping

## Abstract

***Background:*** To investigate maternal-fetal medicine (MFM) physicians' approaches to periviable delivery management and examine whether personal characteristics, practice features, or cognitive traits are associated with these approaches.

***Study Design:*** This was a cross-sectional survey study of Society for Maternal-Fetal Medicine members. Participants were queried regarding recommendations for periviable delivery management based on eight scenarios, as well as personal/practice characteristics and cognitive traits. Responses to scenarios were summarized as “willingness to intervene” and “willingness to recommend termination” scores. We performed a planned sensitivity analysis of the 21-week scenarios, a point considered by some to have clinical equipoise. Top quartile scores were compared with those in the lower three using bivariable and multivariable analyses. Primary analysis assessed association of recommendations with cognitive traits. Secondary analyses included assessment of recommendations with provider personal and practice characteristics.

***Results:*** Of 166 respondents, mean age was 45.5 years (±11.4) and 68.7% were female. Willingness to recommend termination was associated with less willingness to consider cerclage for self or loved one (26.7% vs. 69.4%, *p* < 0.001) and residence in the West or Northeast (*p* = 0.037). Cognitive scores were not associated with recommendations. At 21 weeks, respondents in the top quartile for coping skills were more likely to recommend termination (88% vs. 50%, *p* = 0.017), a finding which persisted after controlling for region (adjusted odds ratio 7.3, 95% confidence interval 1.6–33.0).

***Conclusion:*** MFM physician recommendations for management of pregnancies at risk of periviable delivery were not associated with provider cognitive traits overall, but did vary by provider personal and practice characteristics. In this small, exploratory study, cognitive traits such as coping skills were associated with recommendations, specifically when counseling women at points of clinical equipoise.

## Introduction

The counseling and management of pregnant women at risk of delivering near the limit of viability, or the periviable period, is challenging and highly variable.^1^ Not only is this time period medically complex, but it also can be fraught with anxiety and emotion for both patient and provider.^2^ Infants born in the periviable period, defined as delivery between 20 weeks 0 days and 25 weeks and 6 days of gestational age, suffer the greatest mortality and morbidity of all preterm births. Moreover, women who experience a periviable delivery are nearly six times more likely to have a composite poor maternal outcome than those who deliver at term.^3^ Thus, decision-making in this critical period requires complex counseling and care.

Consensus documents on periviability advocate for expert, unbiased, and comprehensive counseling that includes all possible management options: intervening for fetal benefit, expectant management and declining interventions, and termination of pregnancy.^4^ Despite this recommendation, counseling remains highly variable. Several studies of obstetricians have found that practice setting, personal characteristics, and institutional practice may impact provider decision-making at periviability.^1,5^ Beyond demographic or personal characteristics, provider cognitive traits have also been associated with obstetric care and outcomes. For example, our group's data previously demonstrated that obstetricians' cognitive and affective traits, including tolerance of ambiguity and coping skills, were associated with labor management in term pregnancies.^6,7^

Given these associations between provider factors and obstetrical care, it is possible that provider cognitive traits may also be associated with counseling and decision-making in the periviable period where there are few rules or algorithms to guide management decisions. Due to the rarity of patients at risk for imminent periviable delivery, such decision-making is exceedingly difficult to study in real-time. To our knowledge, no studies have focused on cognitive traits among perinatologists, the obstetric specialists who are often called upon to provide periviability counseling and facilitate patient decision-making. In an effort to fill this gap in our current understanding of periviable practice, we aimed to examine whether maternal-fetal medicine (MFM) physicians' personal characteristics and cognitive traits were associated with their counseling recommendations for women at high risk of periviable delivery.

## Materials and Methods

This was an observational survey-based study conducted from October 2016 to May 2017. During this time period, a web-based survey was sent to Society for Maternal-Fetal Medicine (SMFM) Regular and Fellow members, practicing MFMs and fellows listed on academic websites, and members of the SMFM Members Facebook Page. The study was also listed for 6 months on the SMFM official website listing “surveys of interest.” E-mail invitees received three separate e-mails to complete the survey. Over this time period, 452 direct solicitation e-mails were sent to practicing MFMs and fellows listed on academic websites; the SMFM Members Facebook Page, which requires a validated e-mail address and is cross-checked with a name in the SMFM members registry, had ∼600 members during the study period. Page visits to the SMFM official website are unknown. Institutional Review Board approval from Northwestern University was obtained. On the introduction page of the survey, participants were notified that proceeding to the next page constituted consent.

The self-administered web-based survey instrument queried provider recommendations for periviable delivery management based on two hypothetical scenarios: preterm premature rupture of membranes (PPROM) and advanced cervical dilation. To assess how gestational age influenced provider decision-making, providers were asked for their management recommendations at 19, 21, 23, and 25 weeks. Our primary interest was the decision-making of the perinatologists based on gestational age and presenting complaint. As such, MFMs were asked to offer recommendations in the absence of patient preference.

They received the following prompt: “Your patient is a 25yo nulliparous patient with a singleton, non-anomalous pregnancy. She has no signs of infection or labor. She is well-dated by an 8w ultrasound. She presents with [PPROM/advanced cervical dilation] at the gestational age listed below. You have counseled her about options for management. Given that different patients will make different decisions and it is hard to give recommendations in the absence of patient preference, she asks for your general recommendation in this situation. Assuming all options are available to you, you believe the best course of management is…”

Respondents then selected between the following management options: termination of pregnancy, expectant management with no interventions including no neonatal resuscitation until viability, and some interventions now. If interventions on fetal behalf were selected, additional options appeared. Available options included hospitalization, home bed rest, steroids for fetal lung maturity, antibiotics, tocolytics for the receipt of steroids, long-term tocolysis, and neonatal resuscitation if delivered now. Respondents could choose any number of these interventions.

Responses to vignettes were summarized as separate “willingness to intervene” and “willingness to recommend termination” scores for each respondent. These were calculated by determining the number of times the respondent selected any intervention, expectant management, or termination out of eight possible scenarios. For example, in the case of PPROM, if a participant recommended termination at 19 weeks, expectant management at 21 weeks, and interventions such as steroids and neonatal resuscitation at 23 and 25 weeks, they would receive a score of two for “willingness to intervene” and a score of one for “willingness to recommend termination.” Higher scores for “willingness to intervene” indicated greater tendency to intervene, whereas higher scores for “willingness to recommend termination” indicated greater tendency to recommend termination. Respondent scores were then analyzed in quartiles. Participants who scored in the top quartile scores for intervention and termination were compared with those in the lower three quartiles.

Respondents were additionally surveyed on personal demographics and practice characteristics, including age, gender, race, being a parent, number of years out of fellowship, practice location, and practice setting, as well as questions about cerclage and terminations in their practice. The cognitive traits of ambiguity tolerance and coping skills were assessed using Proactive Coping Inventory (PCI)^8^ and the Multiple Stimulus Types Ambiguity Tolerance-II (MSTAT-II),^9^ validated instruments with previously documented relationships to obstetrical decision-making. For the primary analyses, both scores were assessed as continuous measures given the lack of an established, clinically relevant cutoff point for “normal” or “abnormal” scores. Survey items were pretested with MFM physicians at Northwestern University. Survey data were collected *via* REDCap, a secure web-based application for data capture.

Primary analysis assessed the association of recommendations (*i.e.*, willingness to intervene or willingness to recommend termination scores) with cognitive traits as scored by the PCI and MSTAT-II. Secondary analyses included assessment of recommendations by provider demographics and practice traits. These analyses included all combined gestational ages. Descriptive data were computed with univariate analysis. Bivariate analysis, including Student's *t*-test and chi-squared analyses, was performed as appropriate. A planned sensitivity analysis was performed for 21 weeks as a point of impending clinical equipoise. For the sensitivity analysis, cognitive traits were categorized by quartile with the top quartile (highest scores) representing the most advantageous cognitive traits. Multivariable logistic regression was performed to investigate the independent association of scoring in the top quartile for coping skills with scoring in the top quartile for willingness to terminate, based on the findings described below. Statistical analyses were undertaken using STATA version 11 (StataCorp, College Station, TX).

## Results

Of ∼600 possible MFMs who could have seen the survey advertised on the SMFM Facebook page, 202 individuals responded (33% response rate). Of these, 166 (83%) participants were eligible for inclusion in the final analysis based on availability of complete responses. Thirty-six participants were excluded from analysis for incomplete surveys; those who did not complete the survey did not provide demographic data, and we were thus unable to compare them with MFMs who did complete the survey.

Of the eligible respondents, 68.7% were women, 75.9% identified as white, and mean age was 45.5 years (±11.4). A majority (78.3%) practiced in an urban setting and 80.7% had a full scope of practice, including consultative, inpatient, and deliveries. Although a slight majority of respondents lived in the Northeast and Midwest (54.8%), all regions were roughly evenly represented. A majority of participants (70.9%) had finished fellowship in or after 2000. The participant-reported state gestational age limit for termination was 24 weeks for 51.8% of respondents, and 41.6% stated that the hospital gestational age limit for termination was 24 weeks. Although a minority (10.8%) of respondents worked in a hospital that did not perform terminations, 58.4% of MFMs surveyed did not perform terminations in their current practice.

Regarding the decisions they would make for themselves, 97.0% of MFMs surveyed would consider a termination for themselves or a loved one for maternal indications, and 81.9% would consider a termination for themselves or a loved one if faced with a periviable delivery. Of MFMs surveyed, 88.0% performed cerclage in their current practice, and 57.8% reported that they would consider a cerclage for themselves or a loved one. MFMs who were parents constituted 79.5% of respondents. Table 1 describes the personal and practice characteristics of respondents.

**Table 1.** Participant Characteristics (*n* = 166)

	*n* (%) or mean (SD)
Age (years)	45.5 (±11.4), 30–74 (range)
Female gender	114 (68.7)
Race/Ethnicity^a^	
Non-Hispanic White	126 (75.9)
Non-Hispanic Black	4 (2.4)
Hispanic	13 (7.8)
Asian	17 (10.2)
Other/prefer not to respond	9 (5.4)
Region^b^	
Northeast	47 (28.3)
South	37 (22.3)
Midwest	44 (26.5)
West	38 (22.9)
Practice setting	
Rural	4 (2.4)
Suburban	32 (19.3)
Urban	130 (78.3)
Practice type	
Consultative: includes inpatient but no deliveries	32 (19.3)
Full scope obstetric care, including consultative and deliveries	134 (80.7)
Practice nature	
Academic >50% research	11 (6.6)
Academic >50% clinical practice	97 (58.4)
Academic >50% administrative/education/other	21 (12.7)
Nonacademic group/private practice	19 (11.9)
Current fellow	16 (9.6)
Finished residency	
1970–1979	11 (6.6)
1980–1989	19 (11.4)
1990–1989	30 (18.1)
2000–2009	58 (34.9)
2010–2020	48 (28.9)
Finished fellowship	
1970–1979	2 (1.4)
1980–1989	18 (12.8)
1990–1989	21 (14.9)
2000–2009	46 (32.6)
2010–2020	54 (38.3)
Participant is a parent	132 (79.5)
State upper gestational age limit of termination^c^	
16	0 (0)
18	0 (0)
20	14 (8.4)
22	27 (22.3)
24	86 (51.8)
26	5 (3.0)
No limit	23 (13.9)
Hospital upper gestational age limit of termination	
16	0 (0)
18	1 (0.6)
20	17 (10.2)
22	40 (20.4)
24	69 (41.6)
26	5 (3.0)
No limit	16 (9.6)
Not applicable, hospital does not provide terminations	18 (10.8)
Trained to perform examination-indicated cerclages	160 (96.4)
Perform examination-indicated cerclages in current practice	146 (88.0)
Would consider an examination-indicated cerclage for self or loved one	
No	35 (21.1)
Yes	96 (57.8)
Uncertain	35 (21.1)
Trained to perform terminations	129 (77.7)
Perform terminations in current practice	69 (41.6)
Would consider a termination for self or loved one for maternal indications (*e.g.*, pulmonary hypertension)	
No	1 (0.6)
Yes	161 (97.0)
Uncertain	4 (2.4)
Would consider a termination for self or loved one if faced with risk of periviable delivery	
No	9 (5.4)
Yes	136 (81.9)
Uncertain	21 (12.7)

Data displayed as *n* (%) or mean (SD).

^a^ Totals add up to more than 100% because three respondents selected more than one category for race/ethnicity.

^b^ Regions were specified as follows: *Northeast:* Connecticut, Maine, Massachusetts, New Hampshire, New Jersey, New York, Pennsylvania Rhode Island, Vermont. *South:* Alabama, Arkansas, Delaware, Florida, Georgia, Kentucky, Louisiana, Maryland, Mississippi, North Carolina, Oklahoma, South Carolina, Tennessee, Texas, Virginia, West Virginia. *Midwest:* Illinois, Indiana, Iowa, Kansas, Michigan, Minnesota, Missouri, Ohio, Nebraska, North Dakota, Wisconsin, South Dakota. *West:* Alaska, Arizona, California, Colorado, Hawaii, Idaho, Montana, Nevada, New Mexico, Oregon, Utah, Washington, Wyoming.

^c^ State upper gestational age limit of termination was based on participant report.

SD, standard deviation.

**FIG. 1.**
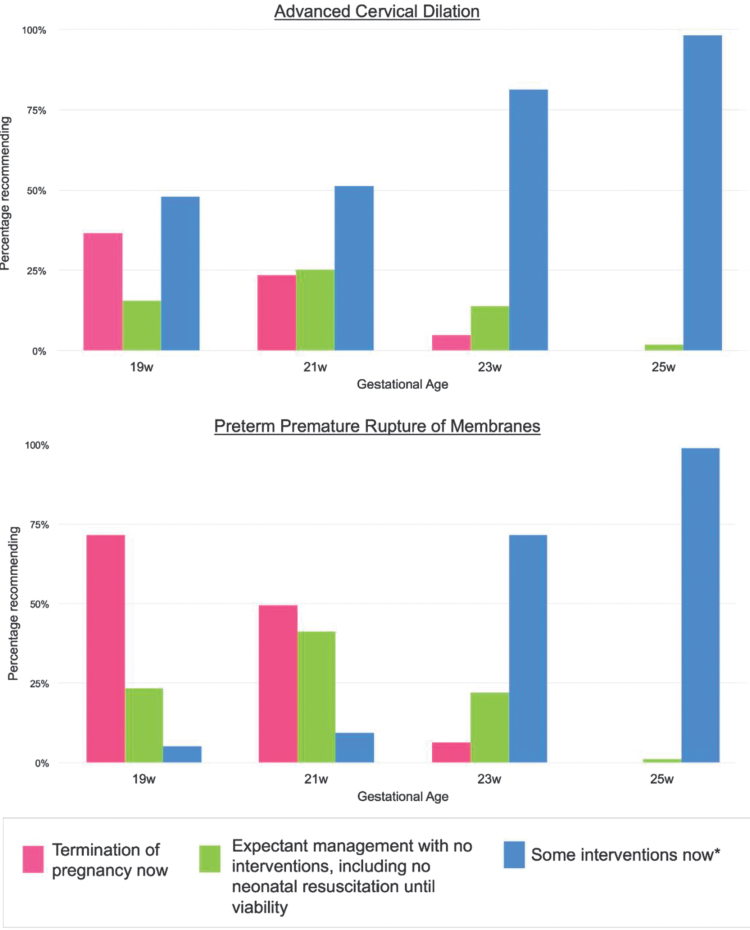
Periviable counseling recommendations by gestational age. *Interventions include hospitalization, home bed rest, steroids for fetal lung maturity, tocolytics for receipt of steroids, long-term tocolytics, antibiotics, cerclage placement and neonatal resuscitation if delivered now.

Figure 1 illustrates recommendations for management of periviable advanced cervical dilation and periviable PPROM. Participants were more likely to intervene at higher gestational ages. In the case of advanced cervical dilation, 51.2% of MFMs recommended intervention at 21 weeks of gestation, and 81.3% recommended intervention at 23 weeks of gestation. Among participants who selected “some interventions now” for the case of advanced cervical dilation, the most common interventions were cerclage placement, hospitalization, antibiotic administration, and home bed rest. For advanced cervical dilation at 19 weeks, cerclage was selected by 78 of the 80 participants who elected intervention, whereas hospitalization was selected by 17, antibiotics by 11, and home bed rest by 9. At 23 weeks, 133 participants selected intervention with 42 selecting cerclage placement, 101 selecting hospitalization, 111 selecting steroids, 45 selecting antibiotics, and 8 selecting home bed rest.

In the case of PPROM, 9.3% of MFMs recommended intervention at 21 weeks of gestation and 71.5% recommended intervention at 23 weeks of gestation. Among participants who selected “some interventions now” for PPROM, the most common intervention was antibiotics. In the case of PPROM at 19 weeks, antibiotic administration was selected by six of the nine participants who opted for interventions, whereas at 23 weeks, 119 respondents selected “some interventions now.” Of those, 111 elected antibiotics, 109 elected steroids for fetal lung maturity, and 102 elected hospitalization.

Recommendation for termination of pregnancy was more frequent at earlier gestational ages (Fig. 1). In the case of advanced cervical dilation, 36.5% of MFMs recommended termination at 19 weeks of gestation, and 23.5% recommended termination at 21 weeks of gestation. Respondents were nearly twice as likely to recommend termination for PPROM at any gestational age than they were for advanced cervical dilation: 71.6% versus 36.5% at 19 weeks and 49.4% versus 23.5% at 21 weeks.

There was no overall association between willingness to intervene and cognitive traits as scored by the PCI and MSTAT-II (Table 2). Similarly, there was no association between willingness to recommend termination and cognitive traits.

**Table 2.** Provider Characteristics by Willingness to Intervene and Willingness to Recommend Termination

	Willingness to intervene: top quartile	Willingness to intervene: lower quartiles	*p*	Willingness to recommend termination: top quartile	Willingness to recommend termination: lower quartiles	*p*
Age, years	46.3 (±13.4)	44.9 (±10.0)	0.460	45.8 (±10.3)	45.3 (±11.9)	0.791
Gender						
Female	41 (63.1)	73 (72.3)	0.282	33 (73.3)	81 (66.9)	0.548
Race/Ethnicity						
Non-Hispanic White	53 (81.5)	73 (72.3)	0.366	33 (73.3)	93 (76.9)	0.070
Asian	4 (6.2)	12 (11.9)		8 (17.8)	8 (6.6)	
Other^a^	8 (12.3)	16 (15.8)		4 (8.9)	20 (16.5)	
Practice setting						
Rural	3 (4.6)	1 (1.0)	0.458	4 (3.3)	0 (0)	0.686
Suburban	12 (18.5)	20 (19.8)		8 (17.8)	24 (19.8)	
Urban	50 (76.9)	80 (79.2)		37 (82.2)	93 (76.9)	
Practice region						
Northeast	14 (21.5)	33 (32.7)	0.128	18 (40)	29 (24.0)	**0.038**
South	19 (29.2)	18 (17.8)		5 (11.1)	32 (26.4)	
Midwest	20 (30.8)	24 (23.8)		9 (20.0)	35 (28.9)	
West	12 (18.5)	26 (25.7)		13 (28.9)	25 (20.7)	
Perform cerclages in current practice						
Yes	59 (90.8)	87 (86.1)	0.515	38 (84.4)	108 (89.3)	0.563
Cerclage for self or loved one						
Yes	50 (76.9)	46 (45.5)	**<0.001**	12 (26.7)	84 (69.4)	**<0.001**
Proactive Coping Inventory	16.26 (±1.94)	15.81 (±1.91)	0.149	15.98 (±1.53)	16.00 (±2.06)	0.947
Multiple Stimulus Types Ambiguity Tolerance-II	62.47 (±11.56)	63.21 (±12.17)	0.701	64.40 (±12.60)	62.36 (±11.63)	0.340

Data displayed as *n* (%) or mean (SD).

Bold indicates significance (*p* < 0.05).

^a^ For the purposes of this table “Other” includes non-Hispanic Black, Black, other, and prefer not to respond. These categories, which in sum were selected by 15.6% of respondents, were combined for statistical analysis.

Secondary outcomes included assessment of recommendations (at all combined gestational ages) with provider personal and practice characteristics (Table 2). MFMs who scored in the top quartile for willingness to intervene were more likely to consider cerclage for themselves or loved ones than MFMs who scored in the lower three quartiles (76.9% vs. 45.5%, *p* < 0.001). Similarly, MFMs who scored in the top quartile for willingness to recommend termination were significantly less likely to consider cerclage for themselves or a loved one than MFMs in the lower three quartiles (26.7% vs. 69.4%, *p* < 0.001). MFMs who scored in the top quartile for willingness to recommend termination were significantly more likely to practice in the West or Northeast (*p* = 0.038). Race, gender, age, practice type, training, performance of cerclage in current practice, and performance of termination in current practice were not associated with provider recommendations.

A sensitivity analysis was performed at 21 weeks of gestation as a point of impending clinical equipoise. MFMs practicing in the Northeast and the West were more likely to recommend termination than intervention at 21 weeks (37.5% termination vs. 25% intervention), while MFMs practicing in the South were more likely to recommend intervention at 21 weeks (9.4% termination vs. 41.7% intervention, *p* = 0.037). Respondents who scored in the top quartile for coping skills were more likely to recommend termination at 21 weeks of gestation than those in bottom quartiles (88% vs. 50%, *p* = 0.017). This finding persisted after controlling for region of practice (adjusted odds ratio 7.3, 95% confidence interval 1.6–33.0). No other differences by provider traits were noted at 21 weeks.

## Discussion

Our study aimed to characterize MFM physicians' counseling and practice patterns for patients at risk of periviable delivery. In this small study, provider cognitive traits, specifically proactive coping and tolerance of ambiguity, were not associated with counseling recommendations. We found that willingness to intervene in the context of periviability was associated with willingness to consider cerclage for self or loved ones. In contrast, providers who were more willing to recommend termination were less willing to consider a cerclage for themselves or a loved one. In addition, MFMs who practiced in the West or Northeast were more likely to recommend termination than MFMs practicing elsewhere. In sensitivity analyses examining a point of clinical equipoise, we identified that these regional differences persisted and that higher coping skills were associated with greater likelihood of scoring in the top quartile for willingness to recommend termination.

Our finding that MFMs who would consider a cerclage for themselves or a loved one were more willing to intervene in a periviable pregnancy adds to data on the ways that provider personal preference may influence counseling recommendations. A survey of obstetricians revealed that the gestational age at which they would want a periviable cesarean for themselves or a loved one was associated with the gestational age at which they would administer steroids.^5^ One plausible explanation is that obstetricians are more likely to recommend a treatment they believe works, whether for themselves, their loved ones, or their patients.

 However, several studies also document that perinatologists are willing to offer a cesarean in the periviable period even if they do not believe that it is beneficial^10^ or if they believe that there is inadequate evidence to support the recommendation.^11^ Although there remains uncertainty that it provides fetal benefit, the rate of cesarean delivery at 23 weeks increased 4.5% between 2012 and 2016 suggesting that factors other than clinical evidence contribute to perinatologists' management decisions.^12^ As has been shown in literature about periviable cesarean, cerclage may be another obstetric procedure where variability in use is linked to factors other than its evidence-based indications.^13^

Our study showed that MFMs practicing in the Northeast and West were more likely to recommend termination than MFMs in other regions. While we instructed respondents to assume that all management options, including termination, were available to them, MFMs may have incorporated the limitations of their current practice into their responses. This may explain why MFMs who practice in more restrictive legal environments, such as the Southeast, were less likely to recommend termination than colleagues in the West and Northeast.

Our finding supports that of McKenzie and Tucker Edmonds, who found that obstetricians practicing in the Northeast were significantly more likely to offer induction for PPROM at 22 weeks than those practicing in other regions.^14^ These findings may have public health implications; in the case of PPROM, which confers maternal risk, differential rates of termination based on geographic residence may lead to rates of maternal morbidity that differ based on where a woman lives.

Given the trend toward earlier obstetric interventions, we chose to examine 21 weeks as a point of impending clinical equipoise. Our overall finding that MFMs in the Northeast and West were more likely to offer termination persisted. However, we also found that respondents who scored in the highest quartile for coping skills were more likely to recommend termination at 21 weeks than those in bottom quartiles. While coping skills are scored on a continuum and there is no clinically relevant cutoff between normal or abnormal scores, scoring in the highest quartile for proactive coping skills among obstetricians has been previously associated with increased likelihood of having patients undergo trial of labor after cesarean (TOLAC).^7^ Similar to TOLAC, providers counseling at periviability must be comfortable discussing risks and benefits of management options and use coping skills in the setting of stress. Since prior studies show that only 41% of obstetricians would even offer an induction after a 22-week PPROM when parents have expressed a desire for palliation,^14^ it may take particularly strong coping skills to be comfortable recommending termination of pregnancy.

Further research is needed to determine whether educational interventions on provider coping skills and the ways that provider personal preference may bias counseling recommendations are an effective means of reducing variation in periviable care. Given our findings on provider's willingness to consider cerclage for themselves or a loved one, additional research could assess the association between the provider being a parent and obstetrical decision-making in the context of periviability.

An important strength of this study is the number of MFMs who participated in cognitive trait testing with validated scales. Previous studies examined between 12 and 94 obstetricians^7,15^; our study had complete data from 166. Another strength is that this study asked perinatologists specifically about termination of pregnancy and their management decisions at earlier gestational ages. Other studies have surveyed obstetricians on management decisions, including induction at 22 weeks gestation^14^; our article assesses management at even earlier gestational ages and includes all methods of pregnancy termination, not just induction.

Several limitations must be considered in interpreting the results of our study. Although we aimed to survey a nationally representative sample of MFMs, findings from an online convenience sample of SMFM members are not generalizable to all MFM physicians. There may be selection bias in which MFMs who responded to the survey were more interested in research; 87.7% of respondents worked in academic medicine. It is possible these respondents were more dedicated to evidence-based guidelines for periviability and that our sample underestimates variation in practice as a result. Those who responded to an online survey may have been younger and more likely to feel comfortable with an online interface. Further, our study population was predominantly female, while other surveys of MFMs have been more balanced or majority male. Finally, while our study represents a larger sample size of MFMs who have participated in cognitive trait testing compared to prior studies, the sample size of overall MFMs is small; due to this small sample size, we were not able to compare responses at each different gestational age and offer only the descriptive responses.

The vignettes themselves had limitations, several of which were by design, yet, nonetheless represent opportunities for future work. First, while many MFMs might engage in shared decision-making with the patient at risk of periviable delivery, the vignettes purposefully omitted patient values and asked physicians to make treatment recommendations in the absence of any knowledge of patient preferences. While the goal of the study was to address provider recommendations and characteristics, we acknowledge that these are preference-sensitive decisions that necessitate shared decision-making in clinical practice. Second, using the terminology “terminate the pregnancy” might have excluded some MFMs who would offer an induction at a periviable gestational age but view induction and pregnancy termination as two separate entities. Finally, our findings are based on physician self-report, which may or may not reflect actual practices.

## Conclusions

Our study revealed new findings regarding provider willingness to consider cerclage for themselves and their subsequent patient counseling. It also highlighted an association between provider coping skills and recommendations for a hypothetical periviable patient at 21 weeks and corroborated findings that geographic region is associated with willingness to recommend termination. As interventions at earlier gestational ages become more frequent, it is important to understand the provider-level factors that underlie some of the variation in care.

## Abbreviations Used

MFM: maternal-fetal medicine
MSTAT-II: Multiple Stimulus Types Ambiguity Tolerance-II
PCI: Proactive Coping Inventory
PPROM: preterm premature rupture of membranes
SD: standard deviation
SMFM: Society for Maternal-Fetal Medicine
TOLAC: trial of labor after cesarean
